# Cardiorenal Interactions in Acute Decompensated Heart Failure: Associations Between Renal Dysfunction, Albuminuria, and Echocardiographic Markers of Myocardial Function

**DOI:** 10.3390/life16040645

**Published:** 2026-04-11

**Authors:** Claudia Andreea Palcău, Livia Florentina Păduraru, Ana Maria Alexandra Stănescu

**Affiliations:** 1 Faculty of Medicine, “Carol Davila” University of Medicine and Pharmacy, 050474 Bucharest, Romania; claudia-andreea.nistor@drd.umfcd.ro (C.A.P.); alexandra.stanescu@umfcd.ro (A.M.A.S.); 2 Department of Cardiology, Elias University Hospital, 011461 Bucharest, Romania; 3 Department of Family Medicine, “Carol Davila” Central Military Emergency University Hospital, 010242 Bucharest, Romania; 4 Academy of Romanian Scientists (AOSR), 050085 Bucharest, Romania; 5 “Emil Palade” Center of Excellence for Young Researchers EP-CEYR, The Academy of Romanian Scientists AOSR, 050085 Bucharest, Romania

**Keywords:** cardiorenal syndrome, acute heart failure, renal dysfunction, echocardiography, E/e′ ratio, longitudinal myocardial function, NT-proBNP, albuminuria

## Abstract

**Background:** Renal dysfunction is common in patients hospitalized with acute decompensated heart failure (ADHF) and represents a key component of cardiorenal syndrome. However, the relationships between renal impairment, cardiorenal biomarkers, and echocardiographic markers of myocardial function remain incompletely characterized in ADHF populations. **Methods:** We conducted a cross-sectional analysis of 144 consecutive patients hospitalized with ADHF. Renal dysfunction was defined as an estimated glomerular filtration rate (eGFR) < 60 mL/min/1.73 m^2^. Clinical, laboratory, and echocardiographic parameters were compared according to renal function. Correlation analyses, multivariable logistic regression, and receiver operating characteristic (ROC) curve analyses were performed to evaluate associations between renal dysfunction, cardiorenal biomarkers, and myocardial functional indices. **Results:** Patients with renal dysfunction were older (*p* = 0.002) and more frequently had diabetes mellitus (*p* = 0.006). Echocardiographic evaluation demonstrated significantly lower systolic mitral annular velocity (S′) (*p* < 0.001) and higher E/e′ ratios (*p* < 0.001) in patients with renal dysfunction, whereas left ventricular ejection fraction (*p* = 0.133) and global longitudinal strain (GLS) (*p* = 0.121) were similar between groups. Log-transformed NT-proBNP and albuminuria were significantly correlated with S′, GLS, and E/e′ (all *p* < 0.001). In multivariable analysis adjusted for clinically relevant confounders, chronic kidney disease (OR 8.16, 95% CI 2.13–31.34; *p* = 0.002) and the E/e′ ratio (OR 2.01, 95% CI 1.52–2.66; *p* < 0.001) remained independently associated with renal dysfunction. ROC analysis showed that E/e′ had the strongest ability to distinguish between patients with and without renal dysfunction (AUC 0.887, 95% CI 0.834–0.941; *p* < 0.001). **Conclusions:** Renal dysfunction in ADHF is associated with echocardiographic markers reflecting impaired longitudinal myocardial function and elevated filling pressure, with E/e′ emerging as the strongest echocardiographic correlate. The integration of echocardiographic parameters with cardiorenal biomarkers may improve the characterization of the cardiorenal profile in patients hospitalized with ADHF.

## 1. Introduction

Heart failure (HF) remains a major global health problem and is associated with substantial morbidity, mortality, and healthcare utilization. Despite advances in pharmacological and device therapies, the prognosis of patients hospitalized with acute decompensated heart failure (ADHF) remains poor, particularly among those with concomitant renal dysfunction [1]. Renal impairment is highly prevalent in HF populations and reflects the complex bidirectional interaction between the heart and kidneys, commonly referred to as cardiorenal syndrome [2].

The coexistence of cardiac and renal dysfunction is associated with worse clinical outcomes, including higher rates of hospitalization and mortality. In large observational cohorts of patients with HF, reduced estimated glomerular filtration rate (eGFR) has consistently been identified as an independent predictor of adverse outcomes [3]. Moreover, renal dysfunction is often accompanied by neurohormonal activation, systemic congestion, and increased intracardiac pressure, all of which contribute to progressive myocardial and renal injury [4].

In addition to reduced glomerular filtration, albuminuria has emerged as an important marker of cardiorenal risk. Increased urinary albumin excretion reflects endothelial dysfunction, microvascular damage, and systemic inflammation. Several large epidemiological studies have demonstrated that albuminuria is independently associated with cardiovascular events and mortality, even in individuals without overt chronic kidney disease (CKD) [5]. In patients with HF, albuminuria has been linked to worse clinical status, higher neurohormonal activation, and increased mortality risk [6,7].

Cardiac biomarkers also play a central role in the evaluation of HF severity and prognosis. Natriuretic peptides, particularly N-terminal pro-B-type natriuretic peptide (NT-proBNP), are widely used for diagnosis and risk stratification in HF. Elevated NT-proBNP levels reflect increased myocardial wall stress and intracardiac pressure overload and have been strongly associated with adverse outcomes across the spectrum of HF phenotypes [8]. NT-proBNP reflects myocardial wall stress and intracardiac pressure overload, serving as a marker of cardiac dysfunction and hemodynamic burden. In contrast, albuminuria reflects renal involvement and is considered a marker of endothelial dysfunction, microvascular injury, and systemic inflammation, thereby capturing the renal and vascular components of the cardiorenal axis.

Echocardiography represents the cornerstone of cardiac imaging in HF and provides essential information regarding myocardial function, cardiac structure, and hemodynamic status. Beyond traditional measures such as left ventricular ejection fraction (LVEF), several echocardiographic parameters have emerged as important markers of myocardial dysfunction and elevated filling pressures. Among these, the ratio of early transmitral inflow velocity to early diastolic mitral annular velocity (E/e′) is widely used as a non-invasive estimate of left ventricular filling pressures and has been shown to correlate with invasive hemodynamic measurements [9].

Similarly, tissue Doppler-derived systolic mitral annular velocity (S′) reflects longitudinal myocardial systolic function and may detect subtle abnormalities in ventricular performance that are not captured by LVEF alone [10]. More recently, global longitudinal strain (GLS) derived from speckle-tracking echocardiography has emerged as a sensitive marker of myocardial dysfunction and has demonstrated prognostic value across multiple cardiovascular conditions, including HF [11].

The interaction between renal dysfunction and myocardial functional parameters remains incompletely understood, particularly across different HF phenotypes. Previous studies have suggested that renal impairment may be associated with increased filling pressures, myocardial fibrosis, and microvascular dysfunction, all of which may influence echocardiographic markers of myocardial performance [12]. However, the relationship between cardiorenal biomarkers and echocardiographic indices of myocardial function in acute HF populations remains insufficiently characterized.

Furthermore, the spectrum of HF includes several phenotypes based on left ventricular ejection fraction, including heart failure with preserved ejection fraction (HFpEF), mildly reduced ejection fraction (HFmrEF), and reduced ejection fraction (HFrEF). These phenotypes differ substantially in terms of pathophysiology, hemodynamic profiles, and underlying myocardial remodeling [13]. Whether the association between renal dysfunction and echocardiographic markers of myocardial dysfunction differs across these phenotypes remains an area of active investigation.

Therefore, the aim of the present study was to investigate the relationship between renal dysfunction, cardiorenal biomarkers, and echocardiographic parameters of myocardial function in patients hospitalized with ADHF. Specifically, we evaluated the association between renal function, albuminuria, and NT-proBNP levels with echocardiographic markers including S′, E/e′, and global longitudinal strain. In addition, we explored whether these relationships differed across HF phenotypes defined by left ventricular ejection fraction.

## 2. Materials and Methods

### 2.1. Study Design and Population

This study represents a cross-sectional analysis of a prospectively assembled cohort of patients hospitalized with acute decompensated heart failure (ADHF) at the Department of Cardiology, Elias University Hospital, Bucharest. Consecutive adult patients admitted with a primary diagnosis of ADHF between July 2025 and November 2025 were screened for eligibility.

For the purposes of the present analysis, only clinical, laboratory, and echocardiographic variables obtained during the index hospitalization were considered. Renal function was assessed using the estimated glomerular filtration rate (eGFR), calculated according to the CKD-EPI equation. Patients were subsequently stratified according to renal function as described below.

Laboratory parameters were measured using standard automated analyzers in the hospital’s central laboratory, according to routine clinical practice. Due to the observational nature of the study, specific instrument models were not recorded. Transthoracic echocardiography was performed by experienced operators using commercially available ultrasound systems, and measurements were obtained in accordance with ASE/EACVI recommendations.

The study was conducted in accordance with the Declaration of Helsinki and approved by the local Ethics Committee of Elias University Hospital (approval number 28072025-2/28 July 2025).

### 2.2. Inclusion and Exclusion Criteria

In total, 144 consecutive patients met the eligibility criteria and were included in the present analysis. The diagnosis of HF was established according to current European Society of Cardiology (ESC) guideline criteria [1,13]. Both patients with newly diagnosed HF and those with a previously documented history of HF were eligible for inclusion.

Patients were eligible if they met the following criteria: age ≥ 18 years, hospitalization with a primary diagnosis of acute decompensated heart failure (ADHF), availability of complete clinical and laboratory data at admission, and availability of a comprehensive transthoracic echocardiographic examination performed during the index hospitalization. In addition, only patients with documented measurements of NT-proBNP and urinary albumin-to-creatinine ratio (ACR) during hospitalization were included.

For the purpose of the present analysis, “complete data” was defined as the availability of all core variables required for the study objectives, including demographic and clinical characteristics, admission laboratory parameters, NT-proBNP, urinary albumin-to-creatinine ratio (ACR), and a transthoracic echocardiographic examination of sufficient quality to allow reliable measurement of predefined myocardial functional parameters. Because the proportion of patients with incomplete datasets was limited and the present study focused on cross-sectional associations based on directly observed variables, no imputation procedures were performed. The proportion of excluded patients due to incomplete data was low and therefore unlikely to significantly influence the overall findings.

Patients were excluded if they had incomplete or missing clinical, laboratory, or echocardiographic data relevant to the study objectives, if echocardiographic examination was not performed during hospitalization, or if echocardiographic image quality did not allow reliable measurement of myocardial function parameters. Patients with known end-stage renal disease requiring chronic dialysis were also excluded. Additionally, patients presenting with acute systemic conditions that could significantly influence renal function independent of HF, such as septic shock or severe systemic infection, were not included in the analysis. Acute systemic conditions were identified based on the clinical diagnosis documented in the medical records at the time of hospitalization, supported by laboratory and imaging findings when applicable.

### 2.3. Clinical and Laboratory Data Collection

Demographic and clinical characteristics were extracted from the electronic medical records. The collected variables included age, sex, smoking status, body mass index category, history of previous hospitalizations for HF, and New York Heart Association (NYHA) functional class at admission.

Information regarding relevant comorbidities was also recorded, including hypertension, diabetes mellitus, atrial fibrillation, coronary heart disease, dyslipidemia, and CKD. Comorbidities were ascertained from the electronic medical records and verified using previously documented diagnoses, prior discharge summaries, chronic medication records, and the clinical evaluation at admission. CKD was defined based on previously documented diagnoses in the medical records and prior clinical evaluations available at the time of hospitalization.

Laboratory parameters obtained at admission included serum creatinine, estimated glomerular filtration rate (eGFR), NT-proBNP, hemoglobin, sodium, potassium, C-reactive protein, leukocyte count, and urinary albumin-to-creatinine ratio (ACR). Due to their skewed distribution, NT-proBNP and ACR values were logarithmically transformed before inclusion in the statistical analysis.

### 2.4. Echocardiographic Assessment

All patients underwent transthoracic echocardiographic examination during hospitalization. Echocardiographic studies were performed according to the recommendations of the American Society of Echocardiography (ASE) and the European Association of Cardiovascular Imaging (EACVI).

Left ventricular ejection fraction (LVEF) was calculated using the Simpson biplane method. Global longitudinal strain (GLS) was obtained by speckle-tracking echocardiography and expressed as the absolute value of longitudinal myocardial deformation.

Systolic mitral annular velocity (S′) was measured using pulsed-wave tissue Doppler imaging at the septal and lateral mitral annulus, and the mean value of the two measurements was used for analysis. The ratio between early transmitral inflow velocity (E) and early diastolic mitral annular velocity (e′) was calculated as E/e′ and used as a non-invasive estimate of left ventricular filling pressures.

Additional echocardiographic parameters included left atrial volume indexed to body surface area, tricuspid annular plane systolic excursion (TAPSE), pulmonary artery systolic pressure (PASP), and the presence and severity of valvular heart disease. Diastolic dysfunction grade was assessed in accordance with current echocardiographic guideline recommendations.

Echocardiographic examinations were performed by two experienced operators in accordance with ASE/EACVI recommendations. All images were subsequently reviewed jointly to ensure consistency of measurements and interpretation. Formal intra- and inter-observer variability were not systematically assessed.

### 2.5. Definition of Renal Dysfunction and Heart Failure Phenotypes

Renal function was assessed using the estimated glomerular filtration rate (eGFR), calculated according to the CKD-EPI equation [14]. Patients were stratified into two groups according to renal function: preserved renal function (eGFR ≥ 60 mL/min/1.73 m^2^) and renal dysfunction (eGFR < 60 mL/min/1.73 m^2^). Renal function classification in the present study was based on the eGFR measured at admission.

In addition, patients were categorized according to HF phenotype based on left ventricular ejection fraction, in accordance with current ESC guidelines [13]: heart failure with preserved ejection fraction (HFpEF; LVEF ≥ 50%), heart failure with mildly reduced ejection fraction (HFmrEF; LVEF 41–49%), and heart failure with reduced ejection fraction (HFrEF; LVEF ≤ 40%).

The association between renal dysfunction, cardiorenal biomarkers, and echocardiographic parameters of myocardial function was subsequently evaluated across the entire study population and within each HF phenotype.

### 2.6. Statistical Analysis

Statistical analyses were performed using IBM SPSS Statistics version 26 (IBM Corp., Armonk, NY, USA). Continuous variables were assessed for normality using visual inspection of histograms and the Shapiro–Wilk test. Normally distributed variables are presented as mean ± standard deviation (SD), whereas non-normally distributed variables are presented as median and interquartile range (IQR). Categorical variables are expressed as counts and percentages.

Comparisons between patients with preserved renal function and those with renal dysfunction were performed using the Mann–Whitney U test for continuous variables and the chi-square test or Fisher’s exact test, as appropriate, for categorical variables.

Comparisons across HF phenotypes (HFpEF, HFmrEF, and HFrEF) were performed using the Kruskal–Wallis test for continuous variables and the chi-square test for categorical variables. Associations between continuous variables were evaluated using Spearman’s rank correlation coefficient. Because urinary albumin-to-creatinine ratio (ACR) and NT-proBNP exhibited markedly right-skewed distributions, both variables were logarithmically transformed (base 10) prior to correlation and regression analyses.

To identify factors associated with renal dysfunction, multivariable logistic regression analysis was performed. Variables included in the multivariable models were selected a priori based on clinical relevance and potential confounding effects and included age, diabetes mellitus, history of chronic kidney disease, systolic blood pressure, atrial fibrillation, and the E/e′ ratio. Results are reported as odds ratios (ORs) with corresponding 95% confidence intervals (CIs).

To explore the potential overlap between echocardiographic parameters and biomarkers reflecting congestion, an additional multivariable model including log-transformed NT-proBNP was constructed. Furthermore, a sensitivity analysis restricted to patients without pre-existing chronic kidney disease was performed to assess whether the observed associations persisted independently of chronic renal impairment.

Receiver operating characteristic (ROC) curve analysis was used to evaluate the ability of echocardiographic parameters to discriminate renal dysfunction. The area under the curve (AUC) with corresponding 95% confidence intervals was calculated. Cut-off values were determined using the Youden index, defined as the maximum value of sensitivity plus specificity minus one. All statistical tests were two-sided, and a *p* value < 0.05 was considered statistically significant.

## 3. Results

### 3.1. Baseline Characteristics of the Study Population

A total of 144 patients hospitalized with ADHF were included in the final analysis. Based on renal function at admission, patients were stratified into two groups: those with preserved renal function (eGFR ≥ 60 mL/min/1.73 m^2^) and those with renal dysfunction (eGFR < 60 mL/min/1.73 m^2^). The baseline demographic and clinical characteristics of the study population are summarized in Table 1.

Patients with renal dysfunction were significantly older than those with preserved renal function (76.3 ± 10.5 vs. 70.2 ± 12.5 years, *p* = 0.002). The proportion of male patients did not differ significantly between the two groups. Likewise, no significant differences were observed with regard to the prevalence of obesity, hypertension, atrial fibrillation, coronary heart disease, or dyslipidemia.

In contrast, diabetes mellitus was significantly more frequent among patients with renal dysfunction compared with those with preserved renal function (60.3% vs. 37.0%, *p* = 0.006). In addition, a history of CKD was significantly more frequent among patients with reduced eGFR at admission compared with those with preserved renal function (92.1% vs. 39.5%, *p* < 0.001).

Regarding laboratory findings, patients with renal dysfunction exhibited higher NT-proBNP concentrations and higher albuminuria levels, reflected by greater median ACR values. Electrolyte profiles were generally comparable between groups, although serum potassium levels were modestly higher among patients with reduced renal function. Hemoglobin concentrations did not differ significantly between the two groups.

Overall, these findings indicate that patients with renal dysfunction were characterized primarily by older age and a higher prevalence of diabetes mellitus, while most other baseline clinical characteristics were broadly comparable between groups.

### 3.2. Echocardiographic Characteristics According to Renal Function

Left ventricular ejection fraction (LVEF) did not differ significantly between patients with preserved renal function and those with renal dysfunction. Similarly, global longitudinal strain (GLS) values were comparable between the two groups.

However, significant differences were observed in parameters reflecting longitudinal myocardial function and left ventricular filling pressures. Specifically, patients with renal dysfunction exhibited lower systolic mitral annular velocity (S′) compared with those with preserved renal function. In addition, the E/e′ ratio was significantly higher in patients with renal dysfunction.

Other echocardiographic parameters, including left atrial volume index (LAVI), tricuspid annular plane systolic excursion (TAPSE), and pulmonary artery systolic pressure (PASP), did not differ significantly between the groups.

Taken together, these results show that patients with renal dysfunction had lower S′ velocities and higher E/e′ ratios, whereas LVEF and GLS values were similar between groups.

Echocardiographic parameters of the study population are summarized in Table 2.

### 3.3. Correlations Between Cardiorenal Biomarkers and Echocardiographic Parameters

Spearman correlation analyses were performed to evaluate the relationships between cardiorenal biomarkers and echocardiographic parameters of myocardial function. The results are presented in Table 3.

Circulating NT-proBNP levels demonstrated significant associations with several echocardiographic variables. In particular, log-transformed NT-proBNP values were inversely correlated with S′ mean (Spearman ρ = −0.538, *p* < 0.001) and GLS (ρ = −0.502, *p* < 0.001). Conversely, NT-proBNP levels showed a positive correlation with the E/e′ ratio (ρ = 0.496, *p* < 0.001).

Similarly, albuminuria, assessed by log-transformed ACR, demonstrated significant associations with echocardiographic measures of myocardial function. ACR values were negatively correlated with S′ mean (ρ = −0.400, *p* < 0.001) and GLS (ρ = −0.348, *p* < 0.001), while a positive correlation was observed with E/e′ (ρ = 0.311, *p* < 0.001).

These analyses demonstrate significant correlations between cardiorenal biomarkers and echocardiographic parameters of myocardial function.

**Figure 1:**
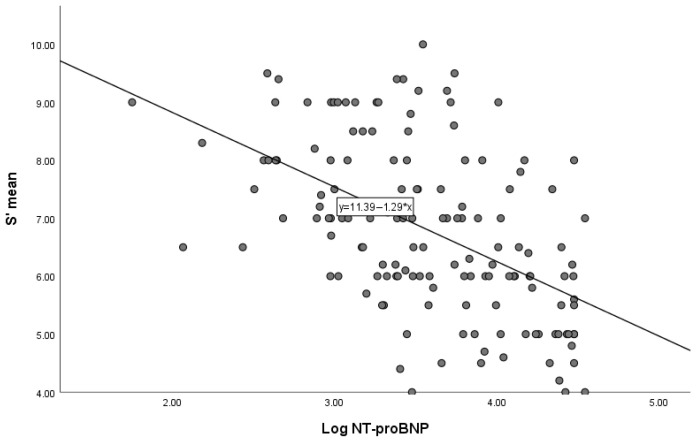
Correlation between NT-proBNP levels and mean systolic mitral annular velocity (S′). Higher NT-proBNP levels were associated with lower S′ values (Spearman ρ = −0.538, *p* < 0.001).

**Figure 2:**
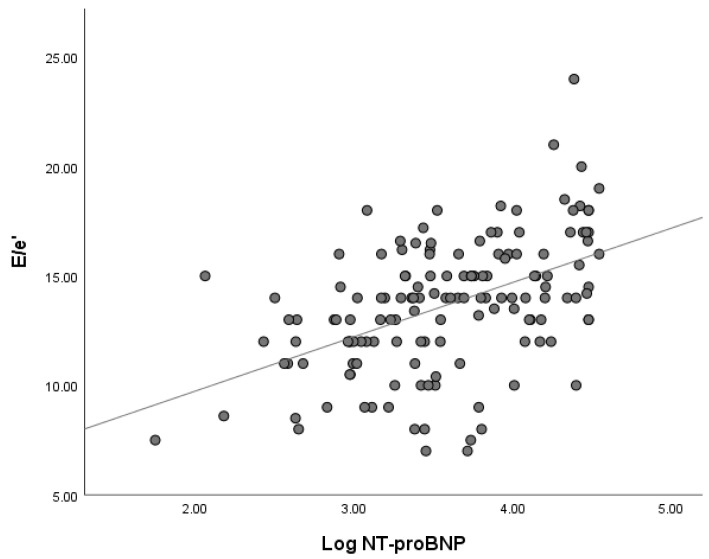
Correlation between NT-proBNP levels and E/e′ ratio. Higher NT-proBNP levels were associated with increased E/e′ values, reflecting elevated left ventricular filling pressures (Spearman ρ = 0.496, *p* < 0.001).

The relationships between NT-proBNP and echocardiographic parameters are illustrated in Figure 1 and Figure 2, which depict the correlations between NT-proBNP and S′ as well as between NT-proBNP and E/e′.

### 3.4. Multivariable Logistic Regression Analysis

To further evaluate factors associated with renal dysfunction, a multivariable logistic regression analysis was performed. The results are presented in Table 4. The overall model was statistically significant (χ^2^ = 98.12, *p* < 0.001) and demonstrated substantial explanatory capacity (Nagelkerke R^2^ = 0.66).

In multivariable logistic regression analysis adjusted for clinically relevant confounders, including age, diabetes mellitus, CKD, systolic blood pressure, and atrial fibrillation, both CKD and the E/e′ ratio remained independently associated with renal dysfunction. Chronic kidney disease was associated with a markedly increased likelihood of renal dysfunction (OR 8.16, 95% CI 2.13–31.34; *p* = 0.002). Importantly, higher E/e′ values were independently associated with renal dysfunction (OR 2.01, 95% CI 1.52–2.66; *p* < 0.001). Atrial fibrillation was also significantly associated with renal dysfunction (OR 0.29, 95% CI 0.09–0.89; *p* = 0.030), whereas age, diabetes mellitus, and systolic blood pressure were not independently associated after multivariable adjustment.

To further assess the potential overlap between echocardiographic parameters and biomarkers reflecting congestion, an additional multivariable model including NT-proBNP was constructed. In this model, CKD (OR 8.42, 95% CI 2.18–32.56; *p* = 0.002), atrial fibrillation (OR 0.26, 95% CI 0.08–0.84; *p* = 0.024), and the E/e′ ratio (OR 2.09, 95% CI 1.55–2.83; *p* < 0.001) remained independently associated with renal dysfunction, whereas log-transformed NT-proBNP was not statistically significant after adjustment (OR 0.67, 95% CI 0.24–1.88; *p* = 0.446).

In a sensitivity analysis restricted to patients without pre-existing CKD, E/e′ remained significantly associated with reduced eGFR at admission (OR 2.45, 95% CI 1.04–5.75; *p* = 0.040), supporting the robustness of the observed association.

### 3.5. Receiver Operating Characteristic (ROC) Curve Analysis

**Figure 3:**
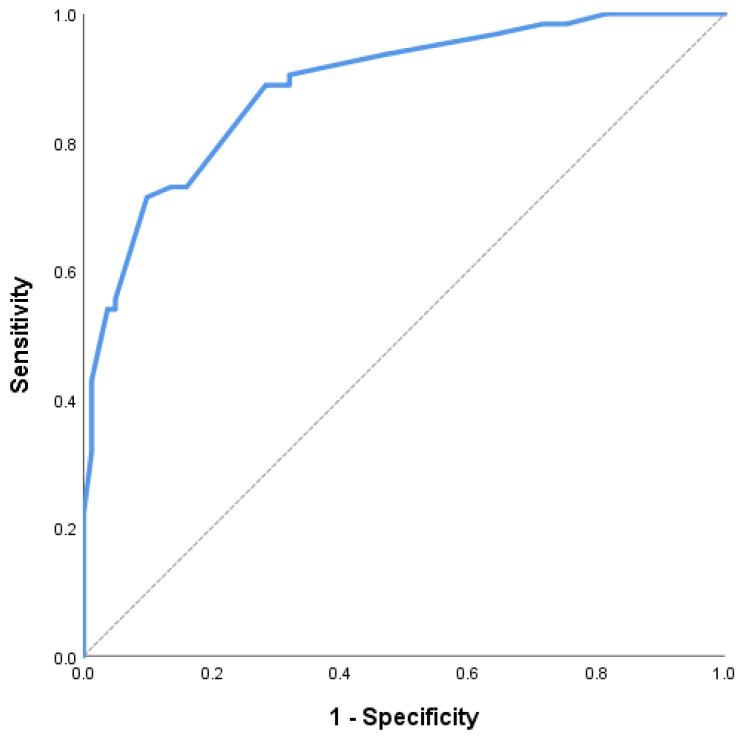
Receiver operating characteristic (ROC) curve of the E/e′ ratio for distinguishing between patients with and without renal dysfunction (eGFR < 60 mL/min/1.73 m^2^). The area under the curve (AUC) was 0.887 (95% CI 0.834–0.941, *p* < 0.001), indicating good diagnostic performance.

**Figure 4:**
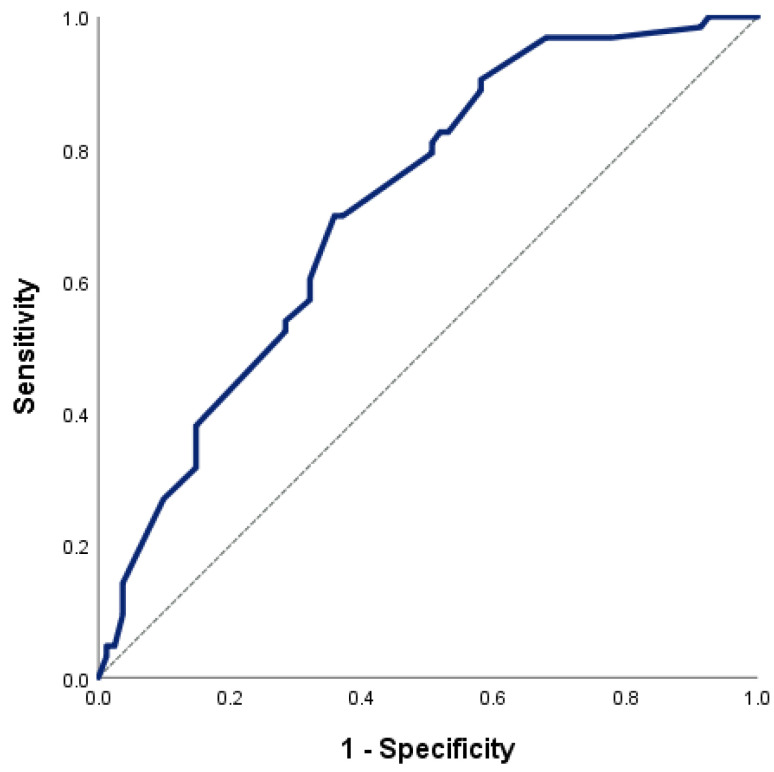
Receiver operating characteristic (ROC) curve of mean systolic mitral annular velocity (S′) for distinguishing between patients with and without renal dysfunction (eGFR < 60 mL/min/1.73 m^2^). The area under the curve (AUC) was 0.715 (95% CI 0.632–0.797, *p* < 0.001), indicating moderate diagnostic performance.

Receiver operating characteristic (ROC) curve analysis was performed to evaluate the ability of echocardiographic parameters to distinguish between patients with and without renal dysfunction. The results of this analysis are summarized in Table 5, while the ROC curves are shown in Figure 3 and Figure 4.

The E/e′ ratio demonstrated excellent ability to distinguish between patients with and without renal dysfunction, with an area under the curve (AUC) of 0.887 (95% CI 0.834–0.941, *p* < 0.001), as illustrated in Figure 3. An optimal cut-off value of 14.0 for the E/e′ ratio was identified, yielding a sensitivity of 88.9% and a specificity of 71.6%.

In comparison, systolic mitral annular velocity (S′) showed moderate discriminative ability, with an AUC of 0.715 (95% CI 0.632–0.797, *p* < 0.001), as shown in Figure 4. An optimal cut-off value of 7.0 cm/s for S′ was identified, yielding a sensitivity of 69.8% and a specificity of 63.0%.

These findings indicate that the E/e′ ratio had greater diagnostic performance for distinguishing between patients with and without renal dysfunction than S′ in this study population.

### 3.6. Cardiorenal Interactions Across Heart Failure Phenotypes

Stratified analyses according to HF phenotype revealed similar patterns of association between renal dysfunction and echocardiographic markers of myocardial function.

Among patients with HFpEF, renal dysfunction was associated with higher ACR values (*p* = 0.028) and higher NT-proBNP levels (*p* = 0.009), as well as with lower S′ velocities (*p* < 0.001) and higher E/e′ ratios (*p* < 0.001). GLS values did not differ significantly between groups.

In the HFmrEF subgroup, renal dysfunction remained significantly associated with lower S′ velocities (*p* = 0.001) and higher E/e′ ratios (*p* < 0.001), whereas no significant differences were observed for ACR, NT-proBNP, or GLS.

Similarly, among patients with HFrEF, renal dysfunction was associated with higher NT-proBNP levels (*p* = 0.004), lower S′ velocities (*p* = 0.004), and higher E/e′ ratios (*p* < 0.001), while GLS values remained comparable between groups.

Overall, lower S′ velocities and higher E/e′ ratios were consistently observed in patients with renal dysfunction across all HF phenotypes.

## 4. Discussion

In this cross-sectional study of patients hospitalized with ADHF, we explored the relationships between renal dysfunction, cardiorenal biomarkers, and echocardiographic parameters of myocardial function. Several important observations emerged. Renal dysfunction was frequent and was associated with older age and a higher prevalence of diabetes mellitus. From an echocardiographic perspective, patients with renal dysfunction were characterized mainly by lower S′ velocities and higher E/e′ ratios, whereas LVEF and GLS were not significantly different between renal function strata. In parallel, both NT-proBNP and albuminuria showed significant associations with echocardiographic markers of myocardial function, and, in multivariable analysis, E/e′, CKD, and atrial fibrillation remained independently associated with renal dysfunction. Furthermore, among the echocardiographic parameters evaluated, E/e′ showed the strongest diagnostic performance for distinguishing between patients with and without renal dysfunction. Notably, a history of CKD was strongly associated with reduced eGFR at admission, supporting the contribution of underlying chronic renal impairment to the observed renal dysfunction in this cohort.

Renal dysfunction is a common and clinically relevant comorbidity in HF and represents a central component of cardiorenal syndrome. Previous studies have shown that impaired kidney function is highly prevalent among patients hospitalized with ADHF and is consistently associated with worse outcomes, including longer hospitalization, greater clinical instability, and higher mortality [1,3,13,15].

Although reduced forward flow has historically been considered a major mechanism of renal dysfunction in HF, more recent evidence supports a broader and more integrated pathophysiological model in which venous congestion, elevated filling pressures, neurohormonal activation, systemic inflammation, and endothelial dysfunction all contribute to renal impairment [16,17].

Our findings are consistent with this concept. In the present cohort, renal dysfunction was not associated with significant differences in conventional measures of global systolic function, such as LVEF, nor with differences in GLS. Instead, the most consistent differences were observed in E/e′ and S′. This pattern is noteworthy because it suggests that the cardiorenal interaction observed during hospitalization for ADHF may be more closely linked to elevated filling pressures and abnormalities in longitudinal myocardial function than to overt changes in global systolic performance.

Among the evaluated parameters, E/e′ emerged as the strongest echocardiographic correlate of renal dysfunction. This observation is in line with previous studies showing that E/e′ correlates with elevated left ventricular filling pressures and is associated with adverse clinical status in HF [9,18,19]. In the present study, E/e′ was higher in patients with renal dysfunction, remained independently associated with renal dysfunction after multivariable adjustment, and showed the highest AUC in ROC analysis. Taken together, these findings support the concept that filling pressure-related congestion is closely linked to renal dysfunction in ADHF.

Importantly, these findings remained robust after adjustment for clinically relevant confounders. In multivariable analysis including age, diabetes mellitus, chronic kidney disease, systolic blood pressure, and atrial fibrillation, the E/e′ ratio remained independently associated with renal dysfunction. This association persisted even after further adjustment for NT-proBNP, suggesting that the relationship between E/e′ and renal dysfunction is not solely explained by biomarker-derived estimates of hemodynamic burden.

Furthermore, in a sensitivity analysis restricted to patients without pre-existing chronic kidney disease, E/e′ remained significantly associated with reduced eGFR at admission. This finding supports the robustness of the observed association and suggests that the relationship between elevated filling pressures and renal dysfunction extends beyond patients with established chronic renal impairment.

At the same time, systolic mitral annular velocity (S′) was consistently lower in patients with renal dysfunction. Tissue Doppler-derived longitudinal systolic indices have long been recognized as sensitive markers of subtle myocardial dysfunction, often preceding overt reductions in LVEF [10]. Prior reports have also suggested that longitudinal myocardial impairment may be particularly relevant in patients with systemic comorbidities, including CKD [20,21,22]. In our cohort, reduced S′ was observed not only in the overall comparison according to renal function but also across HF phenotypes, strengthening the association between renal impairment and longitudinal myocardial dysfunction.

By contrast, GLS did not significantly differ between patients with and without renal dysfunction, despite showing significant correlations with NT-proBNP and ACR. This finding is particularly interesting. GLS is widely recognized as a sensitive marker of myocardial systolic dysfunction and has strong prognostic value across multiple cardiovascular settings [11]. However, its relationship with renal dysfunction appears to be less direct. Some studies have reported associations between impaired GLS and CKD [22], whereas others suggest that GLS may reflect intrinsic myocardial structural abnormalities, including fibrosis and myocardial remodeling, more strongly than congestion-related hemodynamic burden [23,24]. In this context, our results may indicate that, in hospitalized ADHF patients, GLS is less useful than E/e′ or S′ for discriminating renal dysfunction, even though it remains related to overall myocardial functional status. However, this finding should not diminish the broader clinical relevance of GLS, which remains a well-established marker of myocardial dysfunction with important prognostic value across multiple cardiovascular conditions, potentially providing outcome-related information independent of renal function.

These findings are further supported by the observed correlations between cardiorenal biomarkers and echocardiographic parameters of myocardial function. NT-proBNP, a marker of myocardial wall stress and pressure overload, showed significant associations with lower S′, lower GLS, and higher E/e′, indicating a close link between biomarker activation and myocardial functional abnormalities. These results are not unexpected, given the established role of NT-proBNP in reflecting both intracardiac pressure overload and disease severity in HF [25,26].

Importantly, the observed relationship was not limited to natriuretic peptides. Albuminuria, assessed by ACR, was also significantly associated with S′, GLS, and E/e′. This is relevant because albuminuria has increasingly been recognized as a marker of endothelial dysfunction, microvascular injury, and broader cardiovascular risk [27,28]. In the setting of HF, our data suggest that albuminuria is not merely a renal marker but is also linked to echocardiographic manifestations of myocardial dysfunction.

The biomarker findings observed in the present study can be interpreted within the same pathophysiological framework. NT-proBNP, as a marker of myocardial wall stress and pressure overload, likely reflects the hemodynamic burden associated with congestion and elevated filling pressures, which may contribute to renal dysfunction through increased venous pressure and reduced renal perfusion. Likewise, albuminuria may reflect not only renal involvement but also systemic endothelial dysfunction, microvascular injury, and inflammatory activation, thereby representing a broader marker of cardiorenal vulnerability. In this context, the observed associations between these biomarkers and echocardiographic parameters of myocardial function further support the concept of an integrated cardiorenal axis in patients with ADHF.

Another notable aspect of our findings is their relative consistency across HF phenotypes. In the stratified analyses, lower S′ velocities and higher E/e′ ratios were consistently observed in patients with renal dysfunction, regardless of whether they had HFpEF, HFmrEF, or HFrEF. Although the pathophysiological background differs across these phenotypes, this pattern suggests that the association between renal impairment and abnormalities in longitudinal myocardial function and filling pressures may represent a common pathway across the HF spectrum [29,30]. Interestingly, GLS again did not discriminate renal dysfunction within phenotype-specific analyses, which further supports the notion that GLS may capture different aspects of myocardial disease than those most directly related to renal functional decline during acute decompensation.

From a clinical perspective, these findings suggest that echocardiographic parameters reflecting filling pressures, particularly the E/e′ ratio, may serve as useful non-invasive markers for identifying patients at increased risk of renal dysfunction during hospitalization for ADHF. The strong performance of E/e′ in both regression and ROC analyses highlights its potential role as a practical bedside tool for assessing cardiorenal interaction. These findings should, however, be interpreted in the context of shared pathophysiological mechanisms, as both echocardiographic parameters and renal function may reflect the same underlying hemodynamic state rather than a direct causal relationship.

The integration of echocardiographic parameters with cardiorenal biomarkers, including NT-proBNP and albuminuria, may provide a more comprehensive evaluation of disease severity. This combined approach may facilitate early risk stratification, guide closer monitoring of patients with a higher cardiorenal burden, and support more individualized clinical management strategies.

Together, these findings further support the clinical relevance of an integrated cardiorenal assessment. In particular, parameters such as E/e′ and S′, when interpreted alongside NT-proBNP and albuminuria, may provide a more comprehensive evaluation of hemodynamic burden and myocardial dysfunction. This integrated approach may support early risk stratification, guide closer monitoring of patients with a higher cardiorenal burden, and potentially inform more individualized management strategies during hospitalization and after discharge.

The inclusion of clinically relevant confounders in the multivariable models and the consistency of results across sensitivity analyses further strengthen the validity of these findings. Future studies should validate these findings in larger, multicenter cohorts and evaluate whether the combined use of cardiorenal biomarkers and echocardiographic functional parameters may improve risk stratification, monitoring strategies, and management in patients hospitalized with ADHF.

Overall, the present study supports the concept that renal dysfunction in ADHF is closely linked to echocardiographic abnormalities reflecting elevated filling pressures and impaired longitudinal myocardial function. Within this framework, E/e′ appears to occupy a central position, whereas S′ provides additional information on myocardial longitudinal performance. By contrast, GLS, although related to biomarker burden, appears less useful for discriminating renal dysfunction in this specific clinical setting.

## 5. Study Limitations

Several limitations should be acknowledged when interpreting the present findings.

First, this was a single-center study with a relatively modest sample size, which may limit the generalizability of the results. Second, the cross-sectional design precludes establishing causal relationships between renal dysfunction and echocardiographic abnormalities.

Renal function was assessed using a single measurement of eGFR at admission, and dynamic changes in renal function during hospitalization were not evaluated. Therefore, acute fluctuations in kidney function may not have been fully captured. Although several clinically relevant variables were included in the analysis, residual confounding cannot be excluded.

Although information on pre-existing CKD was available, differentiation between CKD, acute kidney injury, and acute-on-chronic renal dysfunction could not be fully and reliably established based on a single measurement at admission.

Echocardiographic parameters were obtained as part of routine clinical assessment by experienced operators; however, intra- and inter-observer variability were not formally evaluated, and measurement variability cannot be excluded.

Potential sources of bias should also be considered. In particular, the exclusion of patients with incomplete datasets may have introduced selection bias, while the exclusion of patients with suboptimal echocardiographic image quality, although methodologically necessary, may limit generalizability.

Despite these limitations, the study provides a detailed characterization of the relationship between renal dysfunction, cardiorenal biomarkers, and echocardiographic parameters of myocardial function in patients hospitalized with ADHF.

## 6. Conclusions

In patients hospitalized with ADHF, renal dysfunction is associated with distinct abnormalities in echocardiographic parameters reflecting impaired longitudinal myocardial function and elevated filling pressure. Among the evaluated echocardiographic markers, the E/e′ ratio showed the strongest association with renal dysfunction, while reduced S′ velocities were also consistently observed in patients with renal dysfunction. In addition, NT-proBNP and albuminuria correlated significantly with echocardiographic indices of myocardial function, further supporting the close interplay between cardiac and renal dysfunction in heart failure.

These findings support the integration of echocardiographic parameters and cardiorenal biomarkers in the comprehensive assessment of patients with acute heart failure and suggest that such an approach may improve characterization of the cardiorenal phenotype in clinical practice.

## Figures and Tables

**Table 1:** Baseline characteristics of the study population according to renal function (eGFR ≥ 60 vs. < 60 mL/min/1.73 m^2^).

Variable	Total (n = 144)	eGFR ≥ 60 mL/min/1.73 m^2^ (n = 81)	eGFR < 60 mL/min/1.73 m^2^ (n = 63)	*p*-Value
Age, years	72.8 ± 11.9	70.2 ± 12.5	76.3 ± 10.5	0.002
Male sex, n (%)	70 (48.6)	35 (43.2)	35 (55.6)	0.141
Obesity, n (%)	80 (55.6)	47 (58.0)	33 (52.4)	0.499
SBP, mmHg (IQR)	130 (115–150)	135 (120–150)	123 (110–150)	0.138
Heart rate, bpm (IQR)	85 (70–100)	90 (73–108.5)	80 (65–90)	0.002
NYHA class				0.173
NYHA II	33 (22.9)	22 (27.2)	11 (17.5)	
NYHA III	41 (28.5)	25 (30.9)	16 (25.4)	
NYHA IV	70 (48.6)	34 (42.0)	36 (57.1)	
HF phenotype, n (%)				0.309
HFpEF,	57 (39.6)	36 (44.4)	21 (33.3)	
HFmrEF	38 (26.4)	18 (22.2)	20 (31.7)	
HFrEF	49 (34.0)	27 (33.3)	22 (34.9)	
Hypertension, n (%)	126 (87.5)	71 (87.7)	55 (87.3)	0.949
Diabetes mellitus, n (%)	68 (47.2)	30 (37.0)	38 (60.3)	0.006
Atrial Fibrillation, n (%)	80 (54.9)	45 (55.6)	35 (54.0)	0.849
Coronary heart disease, n (%)	63 (43.8)	32 (39.5)	31 (49.2)	0.244
Dyslipidemia, n (%)	138 (95.8)	78 (96.3)	60 (95.2)	0.753
CKD, n (%)	90 (62.5)	32 (39.5)	58 (92.1)	<0.001
Hemoglobin g/dL	12.6 ± 2.3	12.9 ± 2.3	12.2 ± 2.3	0.073
Sodium, mmol/L (IQR)	-	138 (134.5–140.2)	139 (135–141.9)	0.328
Potassium mmol/L	4.48 ± 0.69	4.34 ± 0.65	4.66 ± 0.73	0.006
Creatinine, mg/dL (IQR)	-	0.80 (0.70–1.01)	1.70 (1.30–2.20)	<0.001
NT-proBNP, pg/mL (IQR)	-	2650 (1021–6836)	6769 (2450–23032)	<0.001
ACR, mg/g (IQR)	-	58.9 (25.3–163.3)	121.5 (62.2–531.3)	0.002

Data are presented as mean ± SD for normally distributed variables, median (IQR) for non-normally distributed variables, or n (%). Comparisons were performed using the *t*-test, Mann–Whitney U test, χ^2^ test, or Fisher’s exact test, as appropriate. Abbreviations: ACR—albumin-to-creatinine ratio; eGFR—estimated glomerular filtration rate; NT-proBNP—N-terminal pro-B-type natriuretic peptide; NYHA—New York Heart Association; SBP—systolic blood pressure.

**Table 2:** Echocardiographic characteristics of the study population according to renal function (eGFR ≥ 60 vs. <60 mL/min/1.73 m^2^).

Variable	Total (N = 144)	eGFR ≥ 60 mL/min/1.73 m^2^ (n = 81)	eGFR < 60 mL/min/1.73 m^2^ (n = 63)	*p*
LVEF, %	45 (35–55)	45 (35–55)	42 (35–51.5)	0.133
GLS	15 (11–18)	15 (11–18)	14.6 (10.3–16.2)	0.121
S′ mean, cm/s	7 (6–8.5)	7 (6–8.5)	6 (5–7)	<0.001
E/e′	13 (11–15)	12 (10.4–14)	16 (14–17)	<0.001
LAVI, mL/m^2^	41 (36–46)	40 (35–45)	41 (37.5–48)	0.092
TAPSE, mm	21 (18–23)	21 (19–23)	20 (17–22)	0.101
PASP, mmHg	29 (20–40)	26 (20–39)	32 (20–41)	0.371

Data are presented as mean ± SD for normally distributed variables, median (IQR) for non-normally distributed variables, or n (%). Comparisons were performed using the *t*-test, Mann–Whitney U test, χ^2^ test, or Fisher’s exact test, as appropriate.

**Table 3:** Spearman correlations between cardiorenal biomarkers and echocardiographic parameters.

Variable Pair	Spearman ρ	*p*
NT-proBNP_log vs. S′ mean	−0.538	<0.001
NT-proBNP_log vs. E/e′	0.496	<0.001
ACR_log vs. S′ mean	−0.400	<0.001
ACR_log vs. E/e′	0.311	<0.001
NT-proBNP_log vs. GLS	−0.502	<0.001
ACR_log vs. GLS	−0.348	<0.001

Spearman correlation coefficients (ρ) are presented. NT-proBNP and ACR values were log-transformed for analysis. Abbreviations: ACR—albumin-to-creatinine ratio; GLS—global longitudinal strain; S′—systolic mitral annular velocity; E/e′—ratio of early mitral inflow velocity to early diastolic mitral annular velocity.

**Table 4:** Multivariable logistic regression analysis of factors associated with renal dysfunction (eGFR < 60 mL/min/1.73 m^2^) in patients with ADHF.

Variable	OR	95% CI	*p*
Age	1.04	0.99–1.09	0.162
Diabetes mellitus	1.81	0.65–4.99	0.255
CKD	8.16	2.13–31.34	0.002
SBP	0.99	0.98–1.01	0.539
AF	0.29	0.09–0.89	0.030
E/e′	2.01	1.52–2.66	<0.001

Abbreviations: CKD—chronic kidney disease; AF—atrial fibrillation; SBP—systolic blood pressure; OR—odds ratio; CI—confidence interval.

**Table 5:** Diagnostic performance of echocardiographic parameters for identifying renal dysfunction (eGFR < 60 mL/min/1.73 m^2^).

Parameter	AUC	95% CI	*p*	Cut-Off	Sensitivity (%)	Specificity (%)
E/e′	0.887	0.834–0.941	<0.001	14.0	88.9	71.6
S′ mean	0.715	0.632–0.797	<0.001	7.0	69.8	63.0

Cut-off values were determined using the Youden index. The AUC for S′ was calculated using the inverse variable due to the inverse relationship with the outcome. Abbreviations: AUC—area under the curve; CI—confidence interval; S′—systolic mitral annular velocity; E/e′—ratio of early transmitral inflow velocity to early diastolic mitral annular velocity.

## Data Availability

The data presented in this study are available on reasonable request from the corresponding author. The data are not publicly available due to privacy and ethical restrictions.

## Abbreviations

ACR	albumin-to-creatinine ratio
ADHF	acute decompensated heart failure
AUC	area under the curve
CI	confidence interval
CKD	chronic kidney disease
eGFR	estimated glomerular filtration rate
GLS	global longitudinal strain
HF	heart failure
HFpEF	heart failure with preserved ejection fraction
HFmrEF	heart failure with mildly reduced ejection fraction
HFrEF	heart failure with reduced ejection fraction
LAVI	left atrial volume index
LVEF	left ventricular ejection fraction
NT-proBNP	N-terminal pro-B-type natriuretic peptide
PASP	pulmonary artery systolic pressure
ROC	receiver operating characteristic
S′	systolic mitral annular velocity
TAPSE	tricuspid annular plane systolic excursion

## References

[1] McDonagh T.A., Metra M., Adamo M., Gardner R.S., Baumbach A., Böhm M., Burri H., Butler J., Čelutkienė J., Chioncel O. (2021). 2021 ESC Guidelines for the diagnosis and treatment of acute and chronic heart failure. Eur. Heart J..

[2] Martín M., Fernández M., Bacigalupe L.P., Rozado J. (2026). Cardio-Renal Syndrome: Review and New Perspectives. J. Cardioren. Med..

[3] Damman K., Valente M.A.E., Voors A.A., O’Connor C.M., van Veldhuisen D.J., Hillege H.L. (2014). Renal impairment, worsening renal function, and outcome in patients with heart failure: An updated meta-analysis. Eur. Heart J..

[4] Mullens W., Damman K., Harjola V.-P., Mebazaa A., Brunner-La Rocca H.-P., Martens P., Testani J.M., Tang W.H.W., Orso F., Rossignol P. (2019). The use of diuretics in heart failure with congestion—A position statement from the Heart Failure Association of the European Society of Cardiology. Eur. J. Heart Fail..

[5] Matsushita K., van der Velde M., Astor B.C., Woodward M., Levey A.S., de Jong P.E., Coresh J., Gansevoort R.T. (2010). Association of estimated glomerular filtration rate and albuminuria with all-cause and cardiovascular mortality in general population cohorts: A collaborative meta-analysis. Lancet.

[6] Takaoka Y., Horiuchi Y., Strader M., Murray P., Wettersten N. (2025). Albuminuria is associated with worse outcomes in non-diabetics hospitalized with acute heart failure. ESC Heart Fail..

[7] Jackson C.E., Solomon S.D., Gerstein H.C., Zetterstrand S., Olofsson B., Michelson E.L., Granger C.B., Swedberg K., Pfeffer M.A., Yusuf S. (2009). Albuminuria in chronic heart failure: Prevalence and prognostic importance. Lancet.

[8] Tsutsui H., Albert N.M., Coats A.J.S., Anker S.D. (2023). Natriuretic Peptides: Role in the Diagnosis and Management of Heart Failure: A Scientific Statement from the Heart Failure Association of the European Society of Cardiology, Heart Failure Society of America and Japanese Heart Failure Society. J. Card. Fail..

[9] Nagueh S.F., Smiseth O.A., Appleton C.P., Byrd B.F., Dokainish H., Edvardsen T., Flachskampf F.A., Gillebert T.C., Klein A.L., Lancellotti P. (2016). Recommendations for the Evaluation of Left Ventricular Diastolic Function by Echocardiography: An Update from the American Society of Echocardiography and the European Association of Cardiovascular Imaging. J. Am. Soc. Echocardiogr..

[10] Khorshid H., Wadeea B., Sabry E. (2017). Correlation of mitral annular plane systolic excursion (mapse) and tissue doppler peak systolic velocity with left ventricular systolic function. J. Cardiol. Curr. Res..

[11] Kalam K., Otahal P., Marwick T.H. (2014). Prognostic implications of global LV dysfunction: A systematic review and meta-analysis of global longitudinal strain and ejection fraction. Heart.

[12] Damman K., Tang W.H.W., Felker G.M., Lassus J., Zannad F., Krum H., McMurray J.J. (2014). V Current evidence on treatment of patients with chronic systolic heart failure and renal insufficiency: Practical considerations from published data. J. Am. Coll. Cardiol..

[13] McDonagh T.A., Metra M., Adamo M., Gardner R.S., Baumbach A., Böhm M., Burri H., Butler J., Čelutkienė J., Chioncel O. (2023). 2023 Focused Update of the 2021 ESC Guidelines for the diagnosis and treatment of acute and chronic heart failure. Eur. Heart J..

[14] (2024). Kidney Disease: Improving Global Outcomes (KDIGO) CKD Work Group. KDIGO 2024 Clinical Practice Guideline for the Evaluation and Management of Chronic Kidney Disease. Kidney Int..

[15] Francis A., Harhay M.N., Ong A.C.M., Tummalapalli S.L., Ortiz A., Fogo A.B., Fliser D., Roy-Chaudhury P., Fontana M., Nangaku M. (2024). Chronic kidney disease and the global public health agenda: An international consensus. Nat. Rev. Nephrol..

[16] Mocan D., Jipa R., Jipa D.A., Lala R.I., Rasinar F.C., Groza I., Sabau R., Sulea Bratu D., Balta D.F., Cioban S.T. (2025). Unveiling the Systemic Impact of Congestion in Heart Failure: A Narrative Review of Multisystem Pathophysiology and Clinical Implications. J. Cardiovasc. Dev. Dis..

[17] Karimi A., Mathew K., Pourafshar N. (2025). Mechanisms of Congestion in Acute Decompensated Heart Failure. Cardioren. Med..

[18] Luong C.L., Anand V., Padang R., Oh J.K., Arruda-Olson A.M., Bird J.G., Pislaru C., Thaden J.J., Pislaru S.V., Pellikka P.A. (2024). Prognostic Significance of Elevated Left Ventricular Filling Pressures with Exercise: Insights from a Cohort of 14,338 Patients. J. Am. Soc. Echocardiogr..

[19] Zhang X., Li K., Cardoso C., Moctezuma-Ramirez A., Elgalad A. (2024). Interpreting Diastolic Dynamics and Evaluation Through Echocardiography. Life.

[20] Mechal H., Bami A., Haboub M., Choukrani H., Benouna M.E.G., Drighil A., Azzouzi L., Habbal R. (2021). Global Longitudinal Strain of the Left Ventricle in Patients with Chronic Kidney Disease and Hemodialysis. Arch. Cardiovasc. Dis. Suppl..

[21] Panoulas V.F., Sulemane S., Konstantinou K., Bratsas A., Elliott S.J., Dawson D., Frankel A.H., Nihoyannopoulos P. (2015). Early detection of subclinical left ventricular myocardial dysfunction in patients with chronic kidney disease. Eur. Hear. J.-Cardiovasc. Imaging.

[22] Krishnasamy R., Hawley C.M., Stanton T., Pascoe E.M., Campbell K.L., Rossi M., Petchey W., Tan K.-S., Beetham K.S., Coombes J.S. (2015). Left ventricular global longitudinal strain is associated with cardiovascular risk factors and arterial stiffness in chronic kidney disease. BMC Nephrol..

[23] DeVore A.D., McNulty S., Alenezi F., Ersboll M., Vader J.M., Oh J.K., Lin G., Redfield M.M., Lewis G., Semigran M.J. (2017). Impaired left ventricular global longitudinal strain in patients with heart failure with preserved ejection fraction: Insights from the RELAX trial. Eur. J. Heart Fail..

[24] Aikawa T., Kariya T., Yamada K.P., Miyashita S., Bikou O., Tharakan S., Fish K., Ishikawa K. (2020). Impaired left ventricular global longitudinal strain is associated with elevated left ventricular filling pressure after myocardial infarction. Am. J. Physiol. Circ. Physiol..

[25] Januzzi J.L.J., Chen-Tournoux A.A., Christenson R.H., Doros G., Hollander J.E., Levy P.D., Nagurney J.T., Nowak R.M., Pang P.S., Patel D. (2018). N-Terminal Pro-B-Type Natriuretic Peptide in the Emergency Department: The ICON-RELOADED Study. J. Am. Coll. Cardiol..

[26] Chen C., Hsu Y.-C., Chou K.-W., Chang K.-S., Hsu Y.-H., Chiu W.-H., Lee C.-W., Yang P.-S., Chang W.-H., Huang Y.-K. (2024). NT-proBNP point-of-care testing for predicting mortality in end-stage renal disease: A survival analysis. Heliyon.

[27] Claudel S.E., Verma A. (2025). Albuminuria in Cardiovascular, Kidney, and Metabolic Disorders: A State-of-the-Art Review. Circulation.

[28] Matsushita K., Coresh J., Sang Y., Chalmers J., Fox C., Guallar E., Jafar T.H., Jassal S.K., Landman G.W.D., Muntner P. (2015). Estimated glomerular filtration rate and albuminuria for prediction of cardiovascular outcomes: A collaborative meta-analysis of individual participant data. Lancet Diabetes Endocrinol..

[29] Rangaswami J., Bhalla V., Blair J.E.A., Chang T.I., Costa S., Lentine K.L., Lerma E.V., Mezue K., Molitch M., Mullens W. (2019). Cardiorenal Syndrome: Classification, Pathophysiology, Diagnosis, and Treatment Strategies: A Scientific Statement from the American Heart Association. Circulation.

[30] Méndez A.B., Azancot M.A., Olivella A., Soler M.J. (2022). New aspects in cardiorenal syndrome and HFpEF. Clin. Kidney J..

